# Effects of target coverage on local recurrence in stereotactic body radiotherapy for early-stage lung squamous cell carcinoma

**DOI:** 10.1007/s11604-025-01749-x

**Published:** 2025-02-22

**Authors:** Shuou Sudo, Nozomi Kita, Natsuo Tomita, Taiki Takaoka, Dai Okazaki, Masanari Niwa, Akira Torii, Seiya Takano, Masanosuke Oguri, Akane Matsuura, Machiko Ukai, Akio Niimi, Akio Hiwatashi

**Affiliations:** 1 Department of Radiology, Nagoya City University Graduate School of Medical Sciences, 1 Kawasumi, Mizuho-Cho, Mizuho-Ku, Nagoya, Aichi 467-8601 Japan; 2 Department of Respiratory Medicine, Allergy and Clinical Immunology, Nagoya City University Graduate School of Medical Sciences, 1 Kawasumi, Mizuho-Cho, Mizuho-Ku, Nagoya, Aichi 467-8601 Japan

**Keywords:** Stereotactic body radiotherapy, Non-small cell lung cancer, Local recurrence, Coverage

## Abstract

**Background and purpose:**

This study investigated effects of target coverage on local recurrence (LR) in stereotactic body radiotherapy (SBRT) for early-stage lung squamous cell carcinoma (SCC).

**Materials and methods:**

Patients with clinical stage IA1–IIA lung SCC treated with SBRT were included in the analysis. Doses of 48–52 Gy were prescribed to the isocenter of the planning target volume according to the tumor diameter. The primary endpoint was LR. To examine the independent effects of dosimetric factors on LR after adjustment for clinical factors, Fine–Gray model with death as a competing risk was used for evaluation.

**Results:**

Among all 59 patients analyzed, the median follow-up was 42 months. The 3-year LR rate was 24.0%. Univariate analysis of clinical factors showed that biologically effective dose calculated with an *α*/*β* value of 10 (BED_10_) was associated with LR (*p* = 0.033). After adjustment for clinical factors, internal target volume (ITV) Dmean was associated with LR (*p* = 0.049). Subgroup analysis was performed for each prescribed dose group. The results of Fine–Gray model and receiver operating characteristic curve analysis showed that ITV Dmean > 100% of the prescribed dose was the best indicator of preventing LR.

**Conclusions:**

ITV coverage may be particularly important in SBRT for early-stage lung SCC.

## Introduction

Non-small cell lung cancer (NSCLC) is a leading cause of death worldwide. While surgery remains the prevailing gold standard treatment for patients diagnosed with early-stage NSCLC, stereotactic body radiotherapy (SBRT) is an established and effective therapeutic option for medically inoperable patients, offering a local control rate comparable to surgery [1, 2]. However, approximately 15% of early-stage NSCLC patients who undergo SBRT experience local recurrence (LR). LR has a significant impact on prognosis in early-stage NSCLC. We have reported that histological type and tumor size were associated with LR, highlighting a significantly higher incidence of LR in the squamous cell carcinoma (SCC) than adenocarcinoma tumors [3]. Strategy to improve local control in SBRT of early-stage NSCLC includes escalating radiation dose and improving target coverage. Several studies support the prescription of biologically effective dose calculated with an *α*/*β* value of 10 (BED_10_) > 100 Gy as a threshold to achieve a 90% probability of local control with SBRT for early-stage NSCLC [4–8]. In the setting of SBRT for early-stage NSCLC, numerous critical organs, including the hilum, trachea, esophagus, heart, spinal cord, rib bone, and brachial plexus, present limitations of radiation tolerance [9]. It may be necessary to compromise target coverage in close proximity to these critical organs. However, there are few reports that address target coverage, especially in SCC tumors, where LR is more common than in adenocarcinoma. Given the potential clinical benefit of reducing LR by improving target coverage, this study investigated effects of target coverage on LR in SBRT for early-stage lung SCC.

## Material and methods

### Patient selection

Our single institutional database of patients with early-stage NSCLC treated by SBRT was retrospectively analyzed. Inclusion criteria were as follows: (1) clinical Tis-T2bN0M0 according to the 8th TNM classification; (2) treatment with SBRT between February 2004 and September 2018; (3) histologically confirmed SCC; (4) treatment in 4 fractions. Patients with breath hold method were excluded in the analysis. The present study was approved by the Institutional Review Board of Nagoya City University Graduate School of Medical Sciences (approval number: 60-22-0024). This study adhered to the ethical guidelines of the 1964 Declaration of Helsinki and its subsequent revisions. Because this was a retrospective study, the written informed consent was waived, and an opt-out form was provided on the website for those who did not wish to participate.

### Treatment and follow-up

Planning procedures for SBRT are detailed in previous studies. [3, 9, 10]. The treatment planning CT was performed with a slice thickness of 2.5 mm during normal breathing. The internal target volume (ITV) was created to encompass the tumor in all respiratory phases. The planning target volume (PTV) margin was defined as 5 mm in the axial direction and 5–10 mm in the craniocaudal direction. Doses were prescribed to the isocenter of the PTV. Radiation doses were determined based on tumor diameter. Prior to November 2008, doses of 44 Gy, 48 Gy, and 52 Gy were prescribed for tumors with maximum diameters < 1.5 cm, 1.5–3 cm, and > 3 cm, respectively. Beginning in December 2008, doses of 48 Gy, 50 Gy, and 52 Gy were prescribed based on tumor diameter. SBRT was administered twice weekly in 4 fractions, with treatment days scheduled on Mondays and Thursdays or Tuesdays and Fridays based on radiobiologic considerations [11, 12].

CT was performed every 2–3 months until 6 months after SBRT, and then every 6 months for follow-up. ^18^F-fluoro-deoxyglucose positron emission tomography (FDG-PET) and MRI of the brain were performed as needed. The endpoint was defined as LR. LR was essentially diagnosed by CT combined with FDG-PET and/or biopsy.

### Statistical analysis

Clinical parameters, such as age, sex, performance status, smoking, forced expiratory volume in one second (FEV_1_), tumor diameter, histologic types, and BED_10_, were summarized. Dosimetric parameters were evaluated from the dose–volume histogram (DVH) of each individual plan for the gross tumor volume (GTV), ITV, and PTV. Mean dose (*D*_mean_), minimum dose (*D*_min_), maximum dose (*D*_max_), minimum dose to 99% of the volume (D99), D98, D95, D90, D80, D50, and homogeneity index (HI) were calculated for each target volume. Except for HI, all dosimetric factors were expressed as percentages, with 50 Gy serving as the reference value. In the univariate analysis, the Gray test was used to evaluate the association between clinical factors and LR with death as a competing risk, while the log-rank test was used to evaluate the association between clinical factors and overall survival (OS). To examine the independent effects of dosimetric factors on LR after adjustment for clinical factors, Fine–Gray model with death as a competing risk was used for evaluation. Clinical factors with *p*-value < 0.10 in univariate analysis and each dosimetric factor were included in the multivariate model. OS was also evaluated using the Cox proportional hazards model. The optimal threshold for each dose parameter was assessed using receiver operating characteristic (ROC) curves. In our protocol of this study, larger tumor diameters were associated with higher prescription doses as mentioned above; therefore, analysis was conducted by dividing the patients into three subgroups based on the prescribed dose of 48, 50, and 52 Gy. In the subgroup analysis, the coverage was calculated relative to the prescribed dose for each subgroup. The threshold for significance was* p*-value < 0.05. Statistical analysis was performed using EZR (Saitama Medical Center, Jichi Medical University, Saitama, Japan), which is a graphical user interface for R (The R Foundation for Statistical Computing, Vienna, Austria) [13].

## Results

**Fig. 1:**
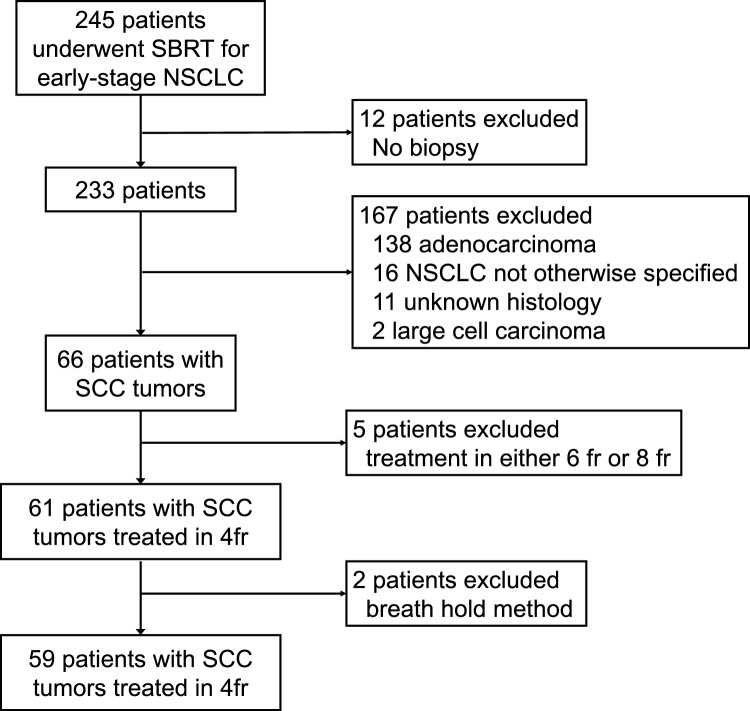
Algorithm for the study cohort

Between February 2004 and September 2018, a total of 245 patients with early-stage NSCLC underwent SBRT at our institution, of which 61 cases were SCC and were treated in 4 fractions. Two cases were excluded due to breath-hold irradiation, resulting in 59 cases being included in the analysis. Figure 1 shows the algorithm of the patient selection.

Patient and treatment characteristics are summarized in Table 1. Fifty-five patients (93%) had a history of smoking. The median follow-up time was 42 months (range, 3–188). The 3-year OS and the progression-free survival were 70.5% [95% confidence interval (CI), 56.4–80.8] and 47.9% (95% CI, 34.3–60.3), respectively. A total of 16 cases of LR were confirmed. The median time between LR and SBRT was 19.5 months (range, 5–123). The 3-year LR rate was 24.0% (95% CI, 13.5–36.1).

**Table 1:** Patient and treatment characteristics

Characteristic	Number or median	% or range
Age (years)	77	58–89
Sex		
Male	51	86%
Female	8	14%
PS		
0, 1	50	85%
2, 3	9	15%
Smoke		
Current or ex-smoker	55	93%
None	2	3%
Missing	2	3%
FEV_1_ (L)	1.62	0.60–3.03
Interstitial pneumonia		
Yes	6	10%
No	53	90%
Tumor diameter (mm)	26	9–50
Tumor location (lobes)		
Upper or middle lobes	40	68%
Lower lobe	19	32%
Total dose (Gy)		
48	21	36%
50	17	29%
52	21	36%
BED_10_		
105.6	21	36%
112.5	17	29%
119.6	21	36%

*PS* performance status, *FEV*_*1*_ forced expiratory volume in one second, *BED*_*10*_ biologically effective dose calculated with an *α*/*β* value of 10

Table 2 shows mean values of target parameters. In most cases, PTV D95 was covered by more than 90% of the prescribed dose. The mean dose for each target parameter was approximately 100% of the prescribed dose.

**Table 2:** Mean values of target parameters

Factors	GTV		ITV		PTV	
	Mean	SD	Mean	SD	Mean	SD
*D*_mean_ (%)	101.60	9.53	100.94	8.78	98.93	7.41
*D*_min_ (%)	93.92	7.63	90.92	7.84	77.95	12.19
D99 (%)	96.72	7.52	94.50	7.08	87.66	7.75
D98 (%)	97.39	7.69	95.67	6.44	89.54	6.47
D95 (%)	98.23	8.03	96.88	6.80	92.32	5.32
D90 (%)	99.01	8.40	97.83	7.23	94.19	5.35
D80 (%)	99.96	8.87	98.98	7.79	96.30	5.94
D50 (%)	101.74	9.75	101.08	9.01	99.53	7.75
*D*_max_ (%)	105.50	10.84	105.69	10.81	106.03	10.67
HI	1.13	0.10	1.17	0.15	1.42	0.42

*GTV* gross tumor volume, *ITV* internal target volume, *PTV* planning target volume, *SD* standard deviation, *Dx* minimum dose to *x*% of the volume, *HI* homogeneity index

Table 3 shows the results of the univariate analyses of clinical factors in relation to LR and OS. In univariate analysis of clinical factors, BED_10_ was associated with LR (*p* = 0.033) and sex, FEV_1_, and tumor diameter was associated with OS (*p* = 0.022, 0.038, and 0.002, respectively).

**Table 3:** Cumulative incidence of local recurrence and overall survival according to patient and treatment characteristics

Factors	*n*	Local recurrence			Overall survival		
		3-Year incidence	95% CI	*p*-Value	3-Year OS	95% CI	*p*-Value
Age				0.17			0.56
≤ 77	31	30.3%	14.8–47.4		73.1%	53.3–85.6	
> 77	28	16.1%	4.8–33.2		66.9%	44.5–82.0	
Sex				0.74			0.022
Male	51	25.4%	14.0–38.6		68.1%	52.7–79.5	
Female	8	14.3%	0.5–49.1		85.7%	33.4–97.9	
PS				0.46			0.15
0,1	50	25.5%	14.0–38.6		74.4%	59.3–84.6	
2,3	9	16.7%	0.4–56.0		50.0%	13.7–78.5	
Smoke				0.49			0.80
Yes	55	21.9%	11.6–34.2		68.1%	53.3–79.2	
No	2	50.0%	0.0–96.0		NA	NA–NA	
FEV_1_				0.52			0.038
≤ 1.62 L	29	22.8%	9.0–40.4		72.9%	51.2–86.1	
> 1.62 L	29	25.9%	11.1–43.5		66.9%	46.0–81.3	
Tumor diameter				0.059			0.002
≤ 26 mm	30	14.2%	4.3–29.8		82.0%	62.0–92.1	
> 26 mm	29	34.9%	17.2–53.3		58.1%	37.0–74.3	
BED_10_				0.033			0.26
≤ 112.5	38	16.5%	6.6–30.3		75.1%	57.5–86.2	
> 112.5	21	38.8%	16.6–60.8		61.5%	35.3–79.7	

*OS* overall survival, *95% CI* 95% confidence interval, *PS* performance status, *FEV*_*1*_ forced expiratory volume in1 s, *BED*_*10*_ biologically effective dose calculated with an *α*/*β* value of 10

**Fig. 2:**
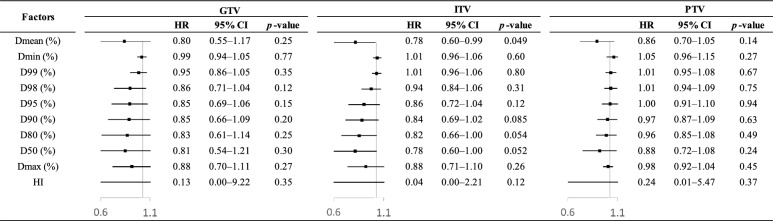
Effects of dosimetric factors on local recurrence after adjustment for clinical factors of *p*-value < 0.10 (i.e., BED_10_ and tumor diameter) in Table 3

**Fig. 3:**
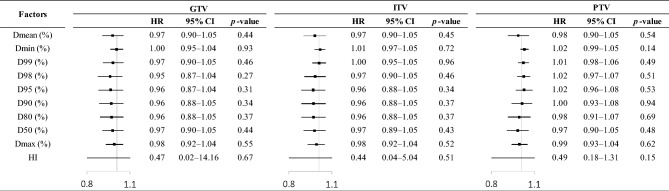
Effects of dosimetric factors on overall survival after adjustment for clinical factors of *p*-value < 0.10 (i.e., sex, FEV_1_, and tumor diameter) in Table 3

Figure 2 shows the effects of dosimetric factors on LR, adjusted for clinical factors of *p*-value < 0.10 in Table 3. ITV Dmean was associated with LR (*p* = 0.049). Figure 3 shows the effects of dosimetric factors on OS, adjusted for clinical factors of *p*-value < 0.10 in Table 3. No dosimetric factors were associated with OS.

The 3-year LR rates were compared between values above and below the ROC threshold using Gray’s test. The area under the curve (AUC) for ITV Dmean was 0.55 (threshold, 96.78%). The 3-year LR rates of the ITV Dmean ≤ 96.78% vs. > 96.78% groups were 20.0% vs. 27.3% (*p* = 0.20), respectively.

**Fig. 4:**
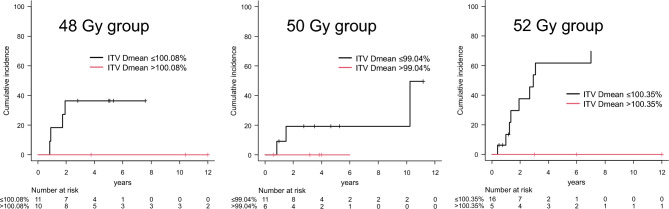
Differences in local recurrence rates according to ITV *D*_mean_ thresholds based on prescribed doses for each subgroup

Table 4 summarizes the results of the subgroup analysis based on the prescribed dose of 48, 50, and 52 Gy. In the subgroup analysis, the coverage was calculated relative to the prescribed dose for each subgroup. The AUC for ITV *D*_mean_ was 0.62 (threshold, 100.08%) in the 48 Gy group, 0.60 (threshold, 99.04%) in the 50 Gy group, and 0.67 (threshold, 100.35%) in the 52 Gy group. The 3-year LR rates were 36.4% vs. 0.0% (*p* = 0.041) for the ITV *D*_mean_ ≤ 100.08% vs. > 100.08% groups in the 48 Gy group, 19.2% vs. 0.0% (*p* = 0.31) for the ITV *D*_mean_ ≤ 99.04% vs. > 99.04% groups in the 50 Gy group, and 53.6% vs. 0.0% (*p* = 0.031) for the ITV Dmean ≤ 100.35% vs. > 100.35% groups in the 52 Gy group (Fig. 4). The results of the Fine–Gray model and ROC curve analysis showed that ITV Dmean > 100% of the prescribed dose was the best indicator of preventing LR.

**Table 4:** Differences in the 3-year local recurrence rates according to ITV *D*_mean_ thresholds based on prescribed doses for each subgroup

Factors	AUC	Threshold	*n*	3-year LR	95% CI	*p*-Value
ITV *D*_mean_ (%) (48 Gy group)	0.62	≤ 100.08	11	36.4%	10.1–64.0	0.041
		> 100.08	10	0.0%	0.0–0.0	
ITV *D*_mean_ (%) (50 Gy group)	0.60	≤ 99.04	11	19.2%	2.5–47.6	0.31
		> 99.04	6	0.0%	0.0–0.0	
ITV *D*_mean_ (%) (52 Gy group)	0.67	≤ 100.35	16	53.6%	22.3–77.2	0.031
		> 100.35	5	0.0%	0.0–0.0	

*AUC* area under the curve, *LR* local recurrence, *95% CI* 95% confidence interval, *ITV* internal target volume

## Discussion

We examined effects of target coverage on LR in SBRT for early-stage lung SCC. ITV Dmean was associated with LR as shown in Fig. 2. The results of the Fine–Gray model and ROC curve analysis showed that ITV *D*_mean _> 100% of the prescribed dose was the best indicator of preventing LR.

In SBRT for early-stage NSCLC, high prescription doses are believed to positively influence treatment outcomes. It is widely accepted that a prescribed BED_10_ > 100 Gy is necessary to achieve over 90% local control. However, there has been ongoing debate regarding the optimal dose due to significant variations in the actual radiation dose received by the target based on the prescription method. A recent study suggested that PTV D95 BED_10_ > 86 Gy and PTV mean BED_10_ > 130 Gy were required to prevent LR [14]. Another research indicated that PTV Dmax BED_10_ < 125 Gy was significantly correlated with LR [15]. In the analysis of SBRT for lung oligometastases, PTV min BED_10_ < 76.6 Gy was significantly correlated with LR [16]. In other treatment modalities, CT-based brachytherapy for cervical cancer, the high-risk CTV D90 was associated with clinical outcomes [17]. However, there are few studies providing specific numerical values for target coverage rates. In our study, ITV *D*_mean_ > 100% of the prescribed dose was the best indicator of preventing LR. Another study focusing on target coverage reported that V95% PTV > 85% was an independent predictor of local control in SBRT for lung oligometastases [18].

The present study showed that ITV mean was most highly correlated with LR. This suggests that ensuring adequate ITV coverage may provide a minimum standard of treatment quality. In clinical practice, when performing SBRT, the proximity of critical structures, such as major blood vessels, esophagus, and heart, may necessitate reduced PTV coverage. In such cases, our results suggest that maintaining adequate ITV coverage could still facilitate high-quality treatment. Even when PTV coverage is compromised, ensuring ITV coverage can lead to effective treatment outcomes. Another study reached similar conclusions, indicating that ITV coverage alone was sufficient for local control [19]. Analyses of oligometastases reported that ITV D90 BED_10_ ≥ 118 Gy and Dmin BED_10_ ≥ 114 Gy improved local control [20]. A previous study further emphasizes that the mean dose to the GTV should be prioritized over PTV coverage [21].

Our study conducted an analysis of cases prescribed at the isocenter. In isocenter prescription, there is a tendency for poorer coverage compared to volumetric dose prescription. Current ESTRO guidelines recommend prescribing over 100 Gy BED_10_ to PTV D95–D99% for achieving a tumor control probability of 90% or higher [22]. With isocenter prescription, there is a possibility that the dose delivered to the tumor may not be guaranteed, and currently, SBRT is performed at our institution using volume dose prescription to PTV D95%.

This study had several limitations. First, it was a retrospective study conducted at a single institution, which has inherent biases associated with retrospective studies. Second, the number of LR cases was small, with only 16 cases, and caution is required when interpreting the results. Large-scale prospective studies are needed to validate the findings of this study. Third, this study used prescribing to isocenter of PTV. As mentioned above, prescribing to D95% of PTV is widely used in SBRT for early-stage NSCLC. At present, our institution also adheres to this approach for SBRT in early-stage NSCLC, and we plan to analyze these data accordingly in future.

## Conclusion

In conclusion, this study investigated effects of target coverage on LR in SBRT for early-stage lung SCC. Among the 59 patients analyzed, 16 developed LR and the median time for onset was 19.5 months after SBRT. The 3-year LR rate was 24.0%. ITV Dmean was associated with LR. The results of the Fine–Gray model and ROC curve analysis showed that ITV *D*_mean_ > 100% of the prescribed dose was the best indicator of preventing LR. This study suggests that ITV coverage was particularly important in SBRT for early-stage lung SCC.
